# Metagenomic discovery of novel enzymes and biosurfactants in a slaughterhouse biofilm microbial community

**DOI:** 10.1038/srep27035

**Published:** 2016-06-08

**Authors:** Stephan Thies, Sonja Christina Rausch, Filip Kovacic, Alexandra Schmidt-Thaler, Susanne Wilhelm, Frank Rosenau, Rolf Daniel, Wolfgang Streit, Jörg Pietruszka, Karl-Erich Jaeger

**Affiliations:** 1 Institut für Molekulare Enzymtechnologie, Heinrich-Heine-Universität Düsseldorf, Jülich, Germany; 2 Institut für Bioorganische Chemie, Heinrich-Heine-Universität Düsseldorf, Jülich, Germany; 3 Zentrum für Peptidpharmazeutika, Universität Ulm, Ulm, Germany; 4 Institut für Mikrobiologie und Genetik, Abteilung für Genomik und Angewandte Mikrobiologie & Göttingen Genomics Laboratory, Georg-August-Universität Göttingen, Germany; 5 Biocenter Klein Flottbek, Department of Microbiology and Biotechnology, University of Hamburg, Hamburg, Germany; 6 Institut für Bio- und Geowissenschaften IBG-1: Biotechnologie, Forschungszentrum Jülich GmbH, Jülich, Germany

## Abstract

DNA derived from environmental samples is a rich source of novel bioactive molecules. The choice of the habitat to be sampled predefines the properties of the biomolecules to be discovered due to the physiological adaptation of the microbial community to the prevailing environmental conditions. We have constructed a metagenomic library in *Escherichia coli* DH10b with environmental DNA (eDNA) isolated from the microbial community of a slaughterhouse drain biofilm consisting mainly of species from the family Flavobacteriaceae. By functional screening of this library we have identified several lipases, proteases and two clones (SA343 and SA354) with biosurfactant and hemolytic activities. Sequence analysis of the respective eDNA fragments and subsequent structure homology modelling identified genes encoding putative N-acyl amino acid synthases with a unique two-domain organisation. The produced biosurfactants were identified by NMR spectroscopy as N-acyltyrosines with N-myristoyltyrosine as the predominant species. Critical micelle concentration and reduction of surface tension were similar to those of chemically synthesised N-myristoyltyrosine. Furthermore, we showed that the newly isolated N-acyltyrosines exhibit antibiotic activity against various bacteria. This is the first report describing the successful application of functional high-throughput screening assays for the identification of biosurfactant producing clones within a metagenomic library.

Metagenomics allow to access novel biocatalysts and metabolites from organisms that are not cultivable^1^,^2^,^3^. In sequence-based approaches, genes are detected using DNA probes or degenerate oligonucleotides derived from known genes encoding the protein family of interest or by homology search of datasets obtained from eDNA deep sequencing^1^,^2^,^4^. In contrast, phenotypic screening approaches apply activity-based assays enabling the discovery of so far unknown proteins belonging to completely novel families. Since the environmental conditions shape the microbial diversity, the choice of the respective habitat is essential for successful mining for novel biocatalysts as shown for cold, hot, and halophilic, habitats^5^,^6^. Furthermore, nutrient availability largely determines the spectrum of enzymes to be identified^7^,^8^.

Regarding phenotypic screening approaches, functional expression of the cognate genes in standard laboratory host strains as well as the availability of efficient screening assays are necessary prerequisites^9^,^10^. Many biotechnological relevant enzymes including hydrolases and oxidoreductases have already been discovered by metagenomic screenings^7^,^11^,^12^. In addition, several secondary metabolites including patellamide D, violaceins, and polytheonamides were successfully isolated in metagenome studies^2^,^13^,^14^. However, the discovery of secondary metabolites still remains challenging, probably due to the demand of proper precursor molecules, functionally interacting auxiliary proteins and the necessity for heterologous expression of large gene clusters^15^,^16^. Another bottleneck is the availability of suitable high throughput screening systems to detect desired natural products^2^,^17^,^18^. Nonetheless, it has been estimated that metagenomic screenings have enormous potential for exploration of novel secondary metabolites^2^,^3^.

Biosurfactants are biologically produced surface active secondary metabolites which can serve as sustainable alternatives for chemically synthesized surfactants^19^. They comprise a structurally diverse group of amphiphilic compounds consisting of hydrophobic fatty acids or fatty acid derivatives and hydrophilic groups, for example sugars, peptides or amino acids^20^. These biosurfactants can be used as detergents and emulsifiers for a variety of agricultural, pharmaceutical and ecological applications^21^. In the last years, many efforts were made to improve the production of already known biosurfactants^22^,^23^,^24^. Furthermore, recently developed high throughput screening methods allowed for the identification of several biosurfactant producing strains from environmental samples^25^,^26^,^27^,^28^, however, to our knowledge, the successful screening of metagenomic libraries for biosurfactants was not yet reported^29^.

In this study, we present the construction and phenotypic screening for lipases, proteases and hemolysins of a metagenomic library obtained from a biofilm isolated from slaughterhouse drain. Furthermore, we describe the successful application of a functional screening assay allowing for the identification of biosurfactant producing clones. A recently constructed expression system^30^ allowed for the efficient expression of the biosurfactant biosynthetic enzymes thereby enabling the identification and initial characterization of the produced biosurfactant.

## Results

### Construction of metagenomic libraries and phylogenetic analysis

**Figure 1:**
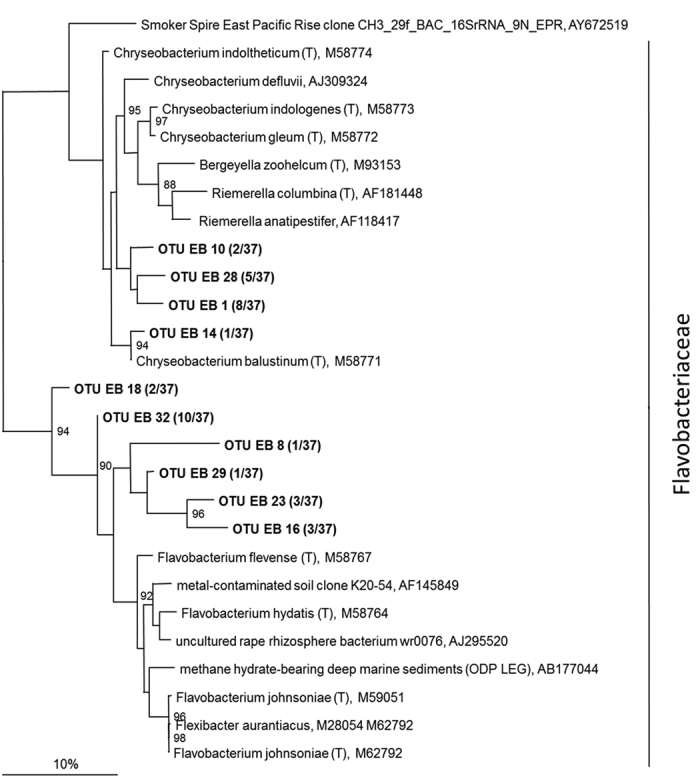
Dendrogram of the 16S rRNA genes identified in the bacterial community of a slaughterhouse biofilm. Phylogenetic relationship of bacterial 16S rRNA gene sequences to the closest known relatives was determined by Maximum-Likelihood analysis. The newly identified sequences were grouped into OTUs (similarity >97%) and are marked in bold. The percentage of bootstrap re-samplings is ≥88 and is indicated at nodes. Numbers in parentheses indicate the amount of sequences associated with the OTU. The scale bar represents the expected number of changes per nucleotide position.

A microbial community living in a biofilm attached to a solid surface of a blood bottom drain in the butchery Frenken Vieh- und Fleisch GmbH (Düren, Germany) was used as eDNA source for the construction of metagenomic libraries. Slaughterhouse material was previously proven to allow for laboratory cultivation of different strains with proteolytic, lipolytic, hemolytic, and biosurfactant activities^31^,^32^,^33^,^34^,^35^. Here, we expected that a microbial community residing in a habitat rich in blood, fats, remnants of animal skin and flesh would be adapted to the efficient degradation of proteins and lipids. The isolated eDNA was used for assessing the phylogenetic diversity of this community by sequence analysis of 16S rRNA genes and the construction of a metagenomic library, which was screened for lipolytic enzymes, proteases, haemolysins and biosurfactants. The biofilm community consisted mainly of *Flavobacterium* sp. and *Chryseobacterium* sp. belonging to the family of Gram-negative, aerobic, rod-shaped Flavobacteriaceae (Fig. 1) with a low G + C genomic content (32–38%).

Metagenomic libraries were constructed in *E. coli* DH10b using eDNA isolated from the bacterial biofilm and cloned into the broad host range shuttle vector pEBP18^30^. The respective clones were grown on LB agar containing tributyrin, skim milk or sheep blood for detection of lipolytic, proteolytic, hemolytic and biosurfactant activities, respectively. The library consisted of 1.7 × 10^5^ clones with >85% containing inserts with an average size of 5.2 kb (size range was 2–10 kb) as determined by analysing 48 randomly selected clones.

### Screening for bioactive molecules

**Figure 2:**
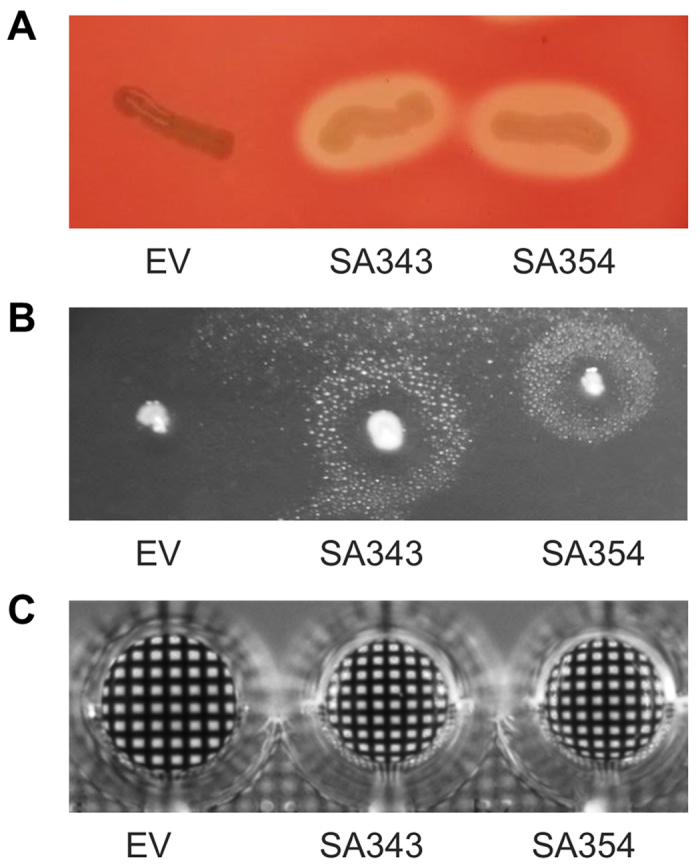
Phenotypic screening of clones from a slaugtherhouse metagenomic library. (**A**) Hemolytic activities detected as halos on blood agar plates. Surface activities detected by (**B**) halo formation with the atomized oil assay and (**C**) an optical distortion of the grid caused by altered surface tension of culture supernatants in microtiter plates. Two *E. coli* DH10b clones expressing genes from environmental DNA fragments SA343 and SA354 were compared with a control strain carrying the empty vector pEBP18 (EV).

The functional screenings of the complete library revealed 15 clones with tributyrin-degrading (TB) lipolytic activity (TB94 - TBIH8), 5 clones with skim milk-degrading (SM) proteolytic activity (SM321 - SMVIIIE12) (Tables S1 and S2, Fig. S1). Additionally, we have identified 2 clones (SA343, SA354) with blood-degrading hemolytic activity (Fig. 2A), which were confirmed as surface active (SA) biosurfactant-producing clones by the atomized oil assay (Fig. 2B). Surface active clones did not produce proteases and phospholipases (data not shown), which are typically considered as hemolysins, thus, biosurfactant production most likely caused hemolysis. We could show that clones SA343 and SA354 produced biosurfactant in liquid culture, as confirmed by the grid assay^36^ (Fig. 2C). To the best of our knowledge, this is the first example of identifying biosurfactant producing clones by screening of a metagenomic library. For further analysis and characterization, approximately 65 mg of crude biosurfactant was produced and isolated from 1 L cultures of *E. coli* DH10b harbouring plasmids pEBP*SA343* or pEBP*SA354*.

### Determination of metagenomic DNA sequences

Plasmids from 22 clones with biological activities detected by screening of the metagenomic library were sequenced and their functions and phylogenetic relationships were predicted from sequence similarity to known genes (Table S2). Apparently, genes of enterobacterial origin were enriched during functional screening in *E. coli*, but the 16S rRNA analysis revealed that the microbial community of the slaughterhouse biofilm consisted predominantly of species from Flavobacteriaceae.

The DNA sequences from clones with proteolytic activity (SM321 - SMVIIIE12) showed 65% to 80% identity with genes from the family of Xanthomonadaceae from the class γ-proteobacteria. At least one open reading frame (ORF) encoding a putative peptidase was identified in each clone thus supporting the results of the functional screening. Detailed analysis showed that clones SM760 and SMVIIIE12 carried identical fragments with six base pair substitutions in non-coding regions. Both clones resembled a truncated version of clone SM679 with which they share 99% sequence identity. Hence, we have identified four novel putative proteases on the three different fragments, since SM321 harbours two ORFs with high similarity to proteolytic enzymes.

Sequence analysis of 15 clones with lipolytic activities revealed that most ORFs showed similarity to genes from α-proteobacteria (TB157, TB303 ORFs 1–3, TB304, TB305, TB306 ORFs 8–9, TB310), β-proteobacteria (TB94, TB307, TB 308, TB312, TB313, TB314, TB I H8) and γ-proteobacteria (TB350). However, genes encoded on eDNA fragments of clones TB303 and TB306 were similar to genes from Flavobacteria in agreement with the phylogenetic analysis of the metagenome-derived eDNA. Each eDNA fragment of the TB clones contained at least one ORF encoding a putative lipolytic enzyme. Accordingly, we have identified 21 novel putative lipase/esterase encoding genes on the eDNA fragments of the 15 lipolytic clones, although some of the sequences were very similar (e.g. TB94 and TB307 are 99% identical).

**Figure 3:**
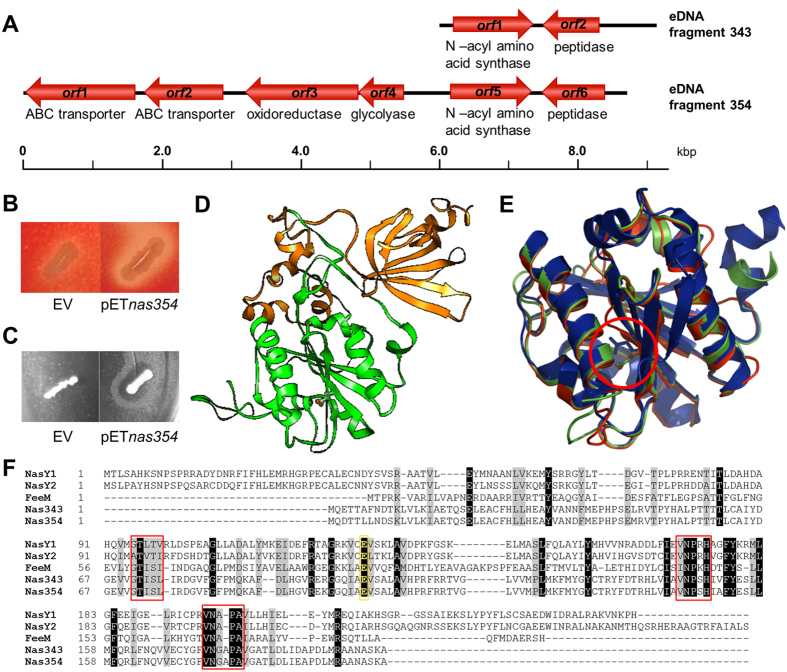
Identification of genes responsible for biosurfactant activity of clones SA343 and SA354. (**A**) Organisation of putative genes identified on eDNA fragments from pEBP*SA343* and pEBP*SA354*. (**B**) Hemolytic activity on blood agar plates, and (**C**) surface activity on LB agar plates determined with the atomized oil assay of *E. coli* BL21(DE3) expressing *nas354* and carrying an empty vector (EV, pET22b). (**D**) Unique two domain architecture of Nas354 (*orf*5 of eDNA fragment 354) modelled with Phyre II server. The model of Nas343 resembles the one of Nas354 (see Fig. S2). In green is shown the N-terminal NAS domain modelled using the crystal structure of FeeM (PDB code 2G0B) and in orange is shown the C-terminal domain modelled using the c-di-GMP-binding protein from *Pseudomonas aeruginosa* (PDB code 1YWU). (**E**) Superimposition of modelled NAS domains of Nas343 (red), Nas354 (green) and FeeM structure (blue) showing the high structural similarity of the three proteins. Structural conservation of the putative catalytic active site glutamate residues shown as sticks is indicated by the red circle. (**F**) Amino acid sequence alignment of NAS domains (residues 1–200) from Nas343 and Nas354 with known type I Nas proteins, FeeM^38^, NasY1 and NasY2^40^. Conserved sequence motifs characteristic for Nas type I proteins are indicated by red boxes. The putative active site glutamate residues are highlighted in yellow. Residues similar and identical in at least four proteins are indicated by grey and black background, respectively.

Clone SA354 contained an 8.8 kb eDNA fragment with parts of the sequence showing up to 88% identity to genomic sequences of the α-proteobacterium *Sinorhizobium*, but none of the six predicted ORFs was similar to known biosurfactant related genes (Fig. 3, Table S2). The two ORFs located on the 3.3 kb fragment obtained from clone SA343 showed similarity to SA354 ORFs with 87% sequence identity (*orf*1 of SA343, *orf*5 of SA354) and 79% sequence identity (*orf*2 of SA343, *orf*6 of SA354) (Fig. 3A, Table S2). The genes encoded by *orf*1 of SA343 and *orf*5 of SA354 showed high similarity (approximately 70% sequence identity in the respective part of the gene) to lysophospholipid acyl transferase genes (Table S2). Parts of *orf*2 from SA343 and *orf*6 from SA354 revealed significant similarity (73% sequence identity) to peptidases. Although *in silico* prediction suggested protease activity, none of the surface active clones showed activity on skim milk agar plates (data not shown). Therefore, the putative acyl transferase genes were considered as the most promising candidates causing surface activity and hemolytic phenotypes of these clones.

### Identification of biosurfactant biosynthesis genes and enzymes

To address the question whether the putative acyl transferase encoding genes are indeed responsible for the observed phenotypes, we constructed a T7-polymerase based expression vector carrying *orf*5 of SA354. Expression of this gene in *E. coli* BL21(DE3) led to the hemolytic and biosurfactant producing phenotypes (Fig. 3B,C). Conclusively, heterologous production of surface active molecules in *E. coli* is presumably catalysed by a single protein encoded by *orf*5 of SA354. This was a striking result, because several genes organised on a contiguous DNA fragment are usually required for the production of heterologous biosurfactant^22^,^37^.

Homology modelling of the putative acyl transferases encoded by *orf*1 of SA343 and *orf*5 of SA354 revealed similarity of their N-terminal domains with N-acyl amino acid synthase FeeM (PDB code 2G0B)^38^ and C-terminal domains with bis-(3′–5′)-cyclic dimeric guanosine monophosphate (c-di-GMP) binding protein PA4608 from *Pseudomonas aeruginosa* (PDB code 1YWU)^39^ (Figs 3D and S3). Remarkably, such a two domain molecular organisation was not found in any previously described protein, neither by sequence similarity search nor by homology modelling.

As the N-acyl amino acid synthase (NAS) domain is likely responsible for the enzymatic activity of the proteins encoded by *orf*1 of SA343 and *orf*5 of SA354, they were designated as Nas343 and Nas354, respectively. The superposition of homology models of N-terminal domains (residues 1–200) of Nas343 and Nas354 with the structure of FeeM^38^ revealed low root-mean-square deviation (RMSD) values (<0.6 Å) as expected for proteins with similar sequence and function. Mainly, these differences can be assigned to the C- and N- terminal parts of each domain, while the main protein folds show no significant differences (Fig. 3E). A sequence alignment of Nas343 and Nas354 with homologous N-acyl amino acid synthases classified them into the type I N-acyl amino acid synthase family characterised by three conserved sequence motifs and a putative active site glutamate residue essential for N-acyl amino acid synthesis activity^40^ (Fig. 3F). In Nas354 and Nas343, we identified Glu103 as the putative active site residues, which showed structural conservation with Glu95 proposed to be the active site residue of FeeM^38^ (Fig. 3E). The substitution of the putative catalytic Glu103 of Nas354 by a functionally unrelated alanine resulted in loss of hemolytic and surface active phenotypes (Fig. S3). These results provide first experimental proof of the function of the conserved glutamate as the catalytic active residue in N-acyl amino acid synthases. Apparently, the catalytic activity of the NAS domain of Nas354 is essential for the hemolytic and biosurfactant producing activities in agreement with previous results which showed that expression of *nas* genes in *E. coli* led to the synthesis of N-acyl derivatives of amino acids^40^,^41^. The previously proposed mechanism of N-acyl amino acid biosynthesis^38^,^42^ suggests that the expression host *E. coli* provides fatty acids linked to acyl-carrier proteins (ACP) which together with Nas343 and Nas354 carry out the biosynthetic reaction.

Homology models of the C-terminal domains (residues 279–394) of Nas343 and Nas354 superimposed with the structure of the PA4608^39^ revealed low RMSD values (<0.5 Å) which indicate a similar function. Residues 200–278 which link the Nas and the c-di-GMP binding domains did not show structural similarity to any known protein structure. Presently, the function of the c-di-GMP binding domain cannot be directly related to the biosurfactant producing activities of Nas354 and Nas343.

### Identification and biophysical properties of the biosurfactant

**Figure 4:**
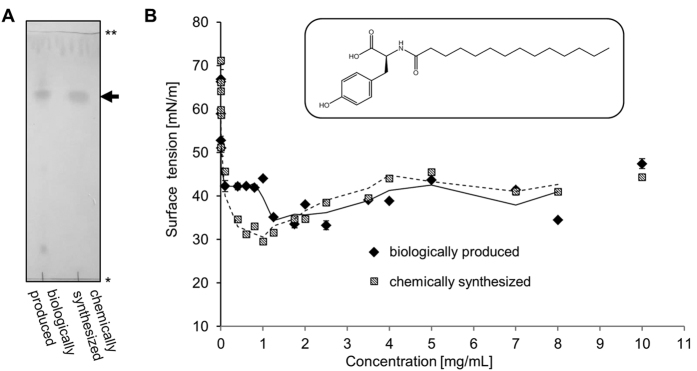
Comparison of chemically synthesized N-myristoyltyrosine with biologically produced N-acyltyrosine. (**A**) Thin layer chromatographic analysis showing similar migration distances (indicated by an arrow) of pure chemically synthesized N-myristoyltyrosine and N-acyltyrosine isolated from culture supernatant of *E. coli* DH10b carrying pEBP*SA354*. Start and solvent fronts are indicated by one and two asterisks, respectively. The TLC plate was stained with iodine vapour. (**B**) Surface tension measurements with chemically synthesized N-myristoyltyrosine and biologically produced N-acyltyrosine show similar properties. Both compounds were dissolved in aqueous solution of NaOH (0.1 M, pH 12). Solid and dotted lines represent trend lines generated with Microsoft Excel2010 to illustrate changes in surface tension dependent on concentration of chemically synthesized N-myristoyltyrosine and biologically produced N-acyltyrosine, respectively. Values represent the mean of three measurements. The structure of the predominant compound of the isolated biosurfactant identified as N-myristoyltyrosine is shown in the coffered box. The structural data were obtained by NMR analysis of biosurfactant purified from liquid cultures of *E. coli* DH10b carrying pEBP*SA343* or pEBP*SA354*.

Biosurfactants produced by clones SA343 and SA354 were identified by thin-layer chromatography (TLC) of culture extracts. For both clones, just one hydrophobic compound with an R_f_ = 0.75 (Figs 4A and S4) was identified. The absence of this compound in the empty vector control indicated that the activities of Nas343 and Nas354 were required for its production. The purified surface-active compound was identified by NMR (see supplementary data) and mass spectrometric (MS) analyses as tyrosine N-acylated with myristic acid (Fig. 4B). Traces of other fatty acids (e.g. unsaturated C_16_ fatty acid) bound to tyrosine were also detected by MS but these compounds were not analysed further. We demonstrated that two novel metagenomic tyrosine NASs, Nas343 and Nas354, enable the synthesis of the biosurfactant N-myristoyltyrosine by *E. coli*.

The biophysical properties N-acyltyrosine purified from clone SA354 including the specific rotation of polarized light, the critical micelle concentration (cmc) and the reduction of surface tension were determined and compared to chemically synthesised N-myristoyl-L-tyrosine (Table 1). Similar values for specific rotation of polarized light for both compounds indicated the presence of *L*- rather than *D*-tyrosine as a component of the biosurfactant. As both biologically and chemically synthesized N-acyltyrosine samples showed low water solubility at neutral pH values, tensiometric characterisation was performed at pH 12 (Fig. 4B). Both compounds drastically reduced the surface tension between the water/air interphase already at low concentrations and they reached a similar constant minimal surface tension at concentrations above 1 mg/mL (Table 1). The cmc’s calculated from tensiometric results (Fig. 4B) were comparable for chemically and biologically synthesised N-myristoyltyrosine (Table 1). These surface active properties confirm that N-acyltyrosine production is responsible for the observed surface active phenotype of the metagenomic clones SA343 and SA354.

### Antimicrobial activity of N-acyltyrosine

**Figure 5:**
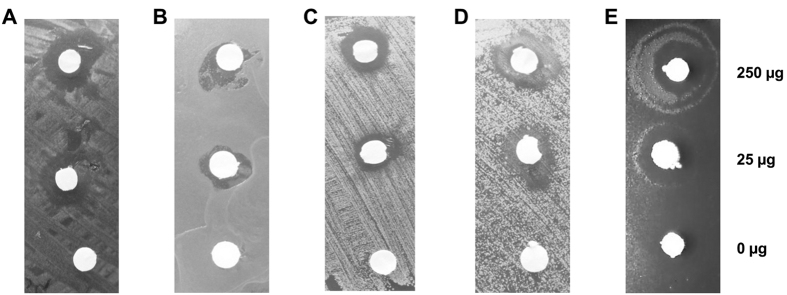
The effect of N-acyltyrosine on the growth of different bacterial strains. A Kirby-Bauer assay was performed with biologically produced N-acyltyrosine at concentrations indicated to the right. Whatman paper discs soaked with N-acyltyrosine dissolved in ethanol were applied to Mueller-Hinton agar plates streaked with: (**A**) *Bacillus subtilis* TEB1030, (**B**) *Sorangium cellulosum* DSM53796, (**C**) *Corynebacterium glutamicum* ATCC13032, (**D**) *Chromobacterium violaceum* CV026. (**E**) Atomized oil assay on Mueller-Hinton agar plates indicates zone of diffusion of N-acyltyrosine under test conditions.

N-acyl amino acids were previously identified as compounds with antibiotic activity against the Gram-positive bacterium *Bacillus subtilis*^40^,^43^; this activity was confirmed for N-acyltyrosine purified from clone SA354 (Fig. 5A). In addition, we have demonstrated inhibitory activity of N-acyltyrosine on the growth of the mycobacterium *Corynebacterium glutamicum*, a Gram-positive bacterium with a cell wall composition different from *B. subtilis*, and the Gram-negative bacteria *Chromobacterium violaceum* CV026 and *Sorangium cellulosum* DSM53796 (Myxobacteria) (Fig. 5B–D). Growth of the γ-proteobacteria *E. coli* DH5α, *P. aeruginosa* PA01, *Serratia marcescens* DSM12481 and *Pseudomonas putida* KT2440 was not affected (data not shown). Antibiotic activity of N-acyltyrosine towards Gram-negative species and Mycobacteria was not reported so far. Although the underlying mechanism is presently unknown, one can speculate that N-acyltyrosine might cause cell membrane disruption as reported for rhamnolipid biosurfactants^44^. This hypothesis is supported by the observation described here and elsewhere that N-acyltyrosine as well as other biosurfactants lyse red blood cells^45^.

## Discussion

Screening of metagenomic libraries offers access to novel bioactive molecules, but requires the combination of a suitable host organism with a functional expression system and an effective screening pipeline. Functional elements to be present include promoters, regulatory sequences and ribosomal binding sites as well as the environment for correct protein folding^46^,^47^ resulting in the so called “different host - different hit” effect^48^.

In this study, *E. coli* DH10b was used as the host for the expression of metagenomics genes encoding both hydrolytic and biosurfactant biosynthetic enzymes. Apparently, this bacterium not only provided suitable cofactors and precursors needed for the synthesis of the target metabolite including compatible acyl carrier proteins required for the synthesis of N-acyl amino acids^38^, but also mechanisms for active or passive secretion of the target metabolite into the culture medium. Furthermore, *E. coli* DH10b showed intrinsic resistance to the produced surface-active compound N-acyltyrosine. It should be noted, however, that this γ-proteobacterial expression system exhibited a clear bias towards expression of proteobacterial genes although the microbial community of the analysed biofilm consisted mostly of Flavobacteria species. Apparently, the functional expression of genes from Flavobacteria is difficult in evolutionary distant *E. coli* as reported previously^49^.

Biosurfactants as metabolites with multiple potential applications represent interesting targets to be identified by metagenomic approaches^2^,^29^. So far, screening methods suitable for the detection of biosurfactant producers in environmental samples were used only to identify novel cultivable bacteria; but they were not applied to screening of metagenomic libraries^26^,^28^. In this study, we have used a newly developed expression system to construct a metagenomic library^30^ which was subsequently subjected to high-throughput screening for biosurfactant producing clones allowing to identify N-acyltyrosine. To the best of our knowledge, this is the first report of a biosurfactant identified by functional screening of a metagenomic library.

N-acyl amino acids have previously been identified as compounds with antibiotic activity towards *B. subtilis*^43^. They were among the first metagenome-derived small molecules identified; however, their surface-active properties were not described so far. They were shown to be synthesized by metagenome-encoded N-acyl amino acid synthases^50^, but until now, only few of these enzymes were identified in cultured bacteria^40^,^51^. The physiological function of N-acyl amino acids and their cognate synthases is still not clear. These enzymes can be incorporated in a metabolic pathway where they catalyse the synthesis of an N-acyl amino acid as an intermediate product^42^, but they may appear as single enzymes as well^38^. Besides that, a genetic link of a subset of these enzymes exists to protein sorting systems belonging to the PEP-CTERM exosortase family^51^. Presumably, genes *nas343* and *nas354* are not located in an operon encoding a biosynthetic pathway. Apparently, also heterologous proteins can accept acyl-ACPs provided by *E. coli* as observed for other acyl-ACP-dependent synthetases^42^,^52^. Although the origin of these genes remains unknown, the high homology of the respective proteins and the generally low overall sequence conservation among NAS type I family members^53^ suggest a close evolutionary relationship of the respective source organisms.

The N-acyl amino acid synthases described here revealed a unique molecular organisation of a NAS domain linked to a putative c-di-GMP binding PilZ domain which has not been observed so far. C-di-GMP is a universal secondary messenger regulating multiple cellular processes in bacteria^54^. PilZ domains with wide phylogenetic distribution were found as a single domain or fused to other domains predicted to have regulatory or transport functions^55^. Binding of c-di-GMP to a PilZ domain usually affects the cellular physiology through modulation of protein-protein or protein-DNA interactions, but a direct effect on the catalytic activity of enzymes involved in alginate and cellulose biosynthesis was also shown^56^,^57^. NasP, a metagenome-derived NAS, was shown to be activated by c-AMP, another widespread bacterial secondary messenger^58^. Therefore, we propose a role of the c-di-GMP binding PilZ domains for the regulation of Nas354 and Nas343 activity; the detailed mechanism needs to be further explored. We observed structural homology of NAS proteins to autoinducer synthetases indicating a function of N-acyl amino acids as cellular messengers^38^ thereby assigning NAS proteins a role as putative regulators of bacterial signaling processes.

The production of N-acyl amino acids is biotechnologically relevant^59^ with N-acyltyrosines being applied in the cosmetic industries^60^,^61^. Comparison of chemically synthesized N-myristoyltyrosine and biologically produced N-acyltyrosine revealed similar surfactant properties although heterogeneity in the natural product was observed for fatty acids bound to tyrosine. Such promiscuity towards fatty acids with different chain lengths and saturation grades is common for biosurfactant synthesizing enzymes^24^,^62^ and may result in the formation of mixed surface films or micelles with altered physical properties^63^. Hence, the here described N-acyl amino acids represent an interesting group of biosurfactants with a remarkably simple biosynthesis and a variety of important biotechnological applications.

## Materials and Methods

### Bacterial strains and growth conditions

The strains and plasmids used in this study are listed in Table 2. In general, bacteria were grown at 37 °C in nutrient rich liquid LB medium or solid LB agar (1.5% w/v) supplemented with kanamycin, 50 mg/L or ampicillin 100 mg/L, if necessary. *E. coli* cells were transformed by the calcium chloride method or by electroporation^64^.

### Recombinant DNA methods

Plasmid DNA was isolated using innuPREP Plasmid Mini Kit (Analytik Jena, Jena Germany) according to the manufacturer’s instructions. DNA ligation, DNA dephosphorylation, restriction endonuclease digestion, and agarose gel electrophoresis were performed according to standard techniques^64^,^65^. Restriction enzymes, T4 DNA ligase, and shrimp alkaline phosphatase (SAP) were purchased from Fermentas (St. Leon-Rot, Germany) or from New England Biolabs (Frankfurt am Main, Germany).

Metagenomic libraries were prepared in pEBP18 as described before^65^. Gene *nas354* was amplified from pEBP*SA354*, using 5′-ATATCATATGCAAGACACCACGTTACTC-3′ and 5′-ATATCTCGAGCTCAGGCTGGGTGGTGTGCA-3′ oligonucleotides and *Pfu* DNA polymerase according to standard protocols. pET*nas354* expression plasmid was constructed by inserting the amplified *nas354* gene in the *Nde*I and *Xba*I restriction sites of pET22b vector. Site directed mutagenesis of *nas354* was performed with QuikChange PCR method using 5′-GGCCAGATTGCCGCAGTGTCGGCCTTG-3′ and 5′-CAAGGCCGACACTGCGGCAATCTGGCC-3′ oligonucleotides and *Pfu* DNA polymerase^66^. Conditions of PCR amplifications were optimized for each primer pair. PCR products were separated on 0.8% (w/v) agarose gels and the desired DNA fragments were purified with the QIAEX II Gel Extraction Kit (QIAGEN, Hilden, Germany) according to manufacturer’s instructions.

### Sequencing

Metagenomic shotgun plasmid libraries were sequenced by the GÖttingen genomics laboratory by a combination of 454 pyrosequencing and Sanger sequencing. The metagenomic plasmid DNA derived from all clones was pooled in equimolar amounts and a 454-shotgun library was generated using the GS FLX Rapid Library Prep kit (Roche Life Science, Mannheim, Germany) following the instructions of the manufacturer. The library was sequenced using a 454 GS-FLX system and Titanium chemistry as recommended by the manufacturer (Roche Life Science). A *de novo* assembly of the reads was performed by using the Roche Newbler assembly software 2.3 (Roche Life Science). The resulting contigs were assigned to the corresponding plasmids by Sanger sequencing of the plasmid inserts ends using BigDye 3.0 chemistry and an ABI3730XL capillary sequencer (Applied Biosystems, Life Technologies GmbH, Darmstadt, Germany). Remaining gaps of the plasmid inserts were closed by PCR-based techniques and Sanger sequencing of the products.

### Bioinformatic analysis

#### Phylogenetic analysis

Bacterial 16S rRNA genes were PCR-amplified according to standard procedures^67^ by using the oligonucleotide primer sets as described^68^,^69^ consisting of 616 V (AGAGTTTGATYMTGGCTCAG) and 1492 R (CGGYTACCTTGTTACGAC) and 140 ng of metagenomic template DNA. The resulting PCR products were ligated into pGEM^®^-T Easy Vector System (Promega, Mannheim, Germany). Transformed competent *E. coli* DH5α cells were grown overnight on LB agar plates and white colonies were randomly selected. Plasmid DNA was extracted and inserts of the correct size were identified by hydrolysis with *Eco*RI. Plasmid preparations were partially sequenced with the (universal) internal 16S rRNA primer GM1F (CCAGCAGCCGCGGTAAT) by Eurofins MWG Operon. Only 16S rRNA sequences with at least 600 bp (37 sequences) were included in phylogenetic analysis. All sequences were edited by Eurofins MWG Operon and compared with DNA sequences in the public domain through BLAST searches^70^. The 16S rRNA gene sequences were compiled and aligned using ARB software^71^. Alignments were then manually checked and sequences grouped into operational taxonomic units (OTUs) exhibiting 97% similarity. Maximum-likelihood trees and 100 bootstrap replicates were constructed with the OTUs and the closest neighbors using PhyML^72^.

#### Database search

Genes encoded on the metagenomic fragments were identified by search for ORFs with Clone Manager software (Scientific & Educational Software, NC, USA). The homology search was performed using the BLAST algorithm available on the NCBI website^70^. The multiple amino acid sequence alignment was generated with the software Clustal Omega^73^.

#### Homology modeling

The three dimensional structure of Nas343 and Nas354 were modelled on the Phyre II server^74^ using the structure of an N-acyl amino acid synthase FeeM (PDB code 2G0B) as a template for the N-terminal domain and the structure of c-di-GMP-binding protein (PDB code 1YWU) as a template for the C-terminal domain. Both template proteins were identified by Phyre II structural homology search algorithms. The UCSF Chimera^75^ and the PyMOL Molecular Graphics System, Version 1.5.0.4 Schrödinger, LLC^76^ were used for structural analysis and visualisation.

### Screening for clones with hydrolytic activity

Protease indicator plates consisted of skim milk agar^77^ with kanamycin (50 μg/mL). Esterase indicator plates contained LB agar supplemented with 1.5% (v/v) tributyrin, 0.15% (w/v) gum arabic and 50 μg/mL kanamycin^78^. Blood agar base (4% w/v) (Carl Roth, Karlsruhe, Germany) dissolved in water supplemented with 5% (v/v) sterilized sheep blood (Fiebig Nähstofftechnik, Idstein-Niederauroff, Germany) and an appropriate antibiotic was used as hemolysis indicator. Clones with hydrolytic activities were identified by the formation of clear halos surrounding the colonies after growth for 24 h at 37 °C on indicator plates followed by storage at 4 °C for several days, if necessary. Phospholipase activity was examined with an egg yolk LB agar plate assay^78^.

### Assays for the detection of biosurfactants

Colonies were grown overnight on LB agar plates supplemented with appropriate antibiotics and inspected for biosurfactant production with the atomized oil assay as previously described using the airbrush “Beginner ESB 100” (Revell) and light paraffin oil (Merck, Darmstadt, Germany)^24^,^79^. The grid assay for detection of surface active substances in liquid media was accomplished in flat bottom microtiter plates positioned onto a grid of squares with a height of 0.8 mm^24^.

### Production and purification of N-acyltyrosine biosurfactant

For the production of N-acyltyrosine in liquid medium, *E. coli* DH10b cells carrying plasmids pEBP*SA343* or pEBPSA354 were grown in Erlenmeyer flasks in 1 L LB medium supplemented with kanamycin (50 μg/mL). The expression cultures were inoculated to an optical density (OD) of OD_580 nm_ = 0.05 and incubated for 18 h at 37 °C under aeration. The culture supernatants were isolated by centrifugation (30 min at 3000 *g*) and filtration, acidified with HCl to pH 3 and left overnight at 4 °C to allow for precipitation of biosurfactants. Precipitates were isolated by centrifugation for 1 h at 8280 *g* at 10 °C, dissolved in water, acidified and centrifuged again as described before. The final precipitate was dissolved in acidified water (pH 3) and the biosurfactant was isolated by three successive extractions with an equal volume of ethyl acetate. The organic phase was evaporated and the remaining solid material was dissolved in ethanol or methanol for subsequent TLC and NMR spectroscopic analysis, respectively.

TLC was performed using Alugram Silica SilG or Polygram SIL G/UV plates (Macherey–Nagel) and a mixture of trichloromethane, methanol, and acetic acid at volume ratio of 65:15:3 as the mobile phase. Spots were detected by illumination with UV light at λ = 365 nm or staining with iodine vapour.

Proteins Nas354 and Nas354_E103A_ were expressed in *E. coli* BL21(DE3) from plasmids pET*nas*354 and pET*nas*354_E103A_, respectively. Cells were grown on LB agar or blood agar plates supplemented with 0.4 mM IPTG and 100 mg/L ampicillin.

### Determination of optical and surface active properties of N-acyltyrosine

The biosurfactant obtained by ethyl acetate extraction was further purified by column chromatography using Merck silica gel 60 (0.063–0.200 mm). The biosurfactant was dissolved in and eluted from the column with a mixture of petroleum ether, ethyl acetate, and acetic acid at volume ratio of 80:20:5.

The surfactant properties of the purified compounds dissolved in 0.1 M NaOH (pH 12) were tested in a concentration range of 1 × 10^−8^ − 10 mg/mL with a micro tensiometer (Kibron Inc, Helsinki, Finnland) utilizing the maximum pull force method^80^,^81^. Each surface tension was measured in triplicate and plotted against the surfactant concentration. The resulting plot was used to determine the cmc and the minimal surface tension using sodium dodecyl sulfate (SDS) as the standard. Optical rotation was measured at 20 °C with a PerkinElmer Polarimeter 241 MC against the sodium D-line.

### NMR spectroscopy

^1^H and ^13^C NMR spectra were recorded at 20 °C with a Bruker Avance/DRX 600 spectrometer in CDCl_3_ with TMS as internal standard. Chemical shifts are given in ppm relative to the Me_4_Si (^1^H: Me_4_Si = 0 ppm) or relative to the resonance of the solvent (^13^C: CDCl_3_ = 77.0 ppm or ^13^C: MeOD = 50.4 ppm) (see supplementary data).

GC-MS (ESI/electron spray ionisation method) was performed with a Hewlett-Packard HP6890 Series-GC-System, 5973 Mass Detector Selective and were recorded by the Competence Center BIOSPEC (Central Institute for Analytics ZEA-3, Forschungszentrum Jülich GmbH, Jülich, Germany).

### Chemical synthesis of N-myristoyl-L-tyrosine

N-Myristoyltyrosine was synthesized as described by Aha^82^ using 1.45 g (8 mmol) of L-tyrosine suspended with 20 mL dichloromethane into a three-necked flask with reflux condenser under nitrogen. 3.1 mL (24.3 mmol) trimethylsilyl chloride was added and the solvent was heated up to reflux for 10 minutes. Hereafter, 3.3 mL (23.8 mmol) of triethylamine were added and the reaction mixture was allowed to reflux for another 10 minutes. Then, the solvent was cooled down over ice and 1.78 g (7.2 mmol) of myristoyl chloride in 5 mL dichloromethane was added slowly. The mixture was stirred for 22 h at room temperature and the reaction subsequently quenched with ice. The white crystalline product was filtrated and washed with distilled water until the filtrate was not acidic anymore. Pure N-myristoyltyrosine was dried under vacuum for 16 h and analysed via NMR (see supplementary data).

### Antibiotic activity assay

Antibiotic activity of isolated biosurfactants was monitored with a modified Kirby Bauer assay^83^. Bacteria obtained from overnight cultures were harvested and dissolved in sterile saline (0.9% w/v NaCl) to a resulting OD_580 nm_ = 0.15. This cell suspension was used to inoculate Mueller-Hinton agar plates with sterile cotton buds. 5 μL of purified N-acyltyrosine dissolved in ethanol (10 mg/mL and 50 mg/mL) were applied to sterile disks of Whatman-Paper (5 mm in diameter), the disks were dried on air and applied to the inoculated Mueller-Hinton agar plates. The antibiotic effect of N-acyltyrosine was estimated after overnight incubation of plates at the optimal growth temperature of each strain as reported by the Leibniz-Institute DSMZ – German Collection of Microorganisms and Cell Cultures. Equally treated disks with pure ethanol were used as negative control.

## Additional Information

**Accession codes:** The sequences of eDNA fragments of clones with hydrolytic and hemolytic activities identified in this study are accessible at NCBI GenBank; accession numbers are provided in Supplementary Table S3. 

**How to cite this article**: Thies, S. *et al*. Metagenomic discovery of novel enzymes and biosurfactants in a slaughterhouse biofilm microbial community. *Sci. Rep*. **6**, 27035; doi: 10.1038/srep27035 (2016).

## Supplementary Material

Supplementary Information

## Figures and Tables

**Table 1:** Physical properties of N-acyltyrosines.

	N-acyl tyrosine biologically produced	N-myristoyl tyrosine chemically synthesized
R_f_-value	0.75	0.75
specific rotation of polarized light	[α]D20 °C = +2.1	[α]D20 °C = +2.034
cmc (pH 12)	0.114% (w/v)	0.063% (w/v)
min. surface tension (pH 12)	34 mN/m	30 mN/m

**Table 2:** Strains and plasmids used in this study.

Strains	Genotype	Reference
*Bacillus subtilis* TEB1030 Marburg 168	wild-type	84
*Chromobacterium violaceum* CV026	variant of ATCC31532 deficient for violacein production	85
*Corynebacterium glutamicum* ATCC13032	wild-type	86
*Escherichia coli* BL21(DE3)	*F*- *ompT hsdSB* (*rB*-*mB*-) *gal dcm* (DE3)	87
*Escherichia coli* DH10b	*F*- *mcrA* Δ(*mrr-hsdRMS-mcrBC*) *Φ80lacZΔM15 ΔlacX74 endA1 recA1 deoR* Δ(*ara,leu*)*7697 araD139 galU galK nupG rpsL λ*-	88
*Escherichia coli* DH5α	F- φ80*lacZ*ΔM15 Δ(lacZYA-*arg*F) U169 *recA*1 *end*A1 *hsdR17* (rk-, mk*+) phoA sup*E44 λ- *thi*-1 *gyrA*96 *relA*1	89
*Pseudomonas aeruginosa* PAO1	wild-type	90
*Pseudomonas putida* KT2440	wild-type	91
*Serratia marcescens* DSM12481	wild-type	92,93
*Sorangium cellulosum* DSM53796	wild-type	94
**Plasmids**	**Genotype**	**Source/reference**
pEBP18	*amyE’ ’amyE* ColE1 cos gfpmut3* Km^R^ Cm^R^ MCS *minR*’ P_T7_ P_xyl_	30
pEBP-*SM321 – SMVIIIE12*	pEBP18 containing fragments of metagenomic DNA of proteolytic clones listed in the table S1	This work
pEBP-*TB94 – TB I H8*	pEBP18 containing fragments of metagenomic DNA from lipolytic clones listed in the table S1	This work
pEBP-*SA343*	pEBP18 containing a 3.3 kb fragment of metagenomic DNA inclusive *nas343* gene	This work
pEBP-*SA354*	pEBP18 containing a 8.8 kb fragment of metagenomic DNA inclusive *nas354* gene	This work
pET22b	*ColE1 P*_*T7 *,_φ10*lacIq, Amp*^*R*^	EMD Millipore
pET*nas354*	pET22b containing the *nas354* gene	This work
pET*nas354*^E103A^	pET22b containing the *nas354* gene with Glu103 substituted by Ala	This work
pGEM^®^-T Easy	Amp^R^, *lacZ*	Promega
