# Supplementary material for: Metagenomic discovery of novel enzymes and biosurfactants in a slaughterhouse biofilm microbial community
Source: Sci Rep. 2016 Jun 8;6:27035. doi: 10.1038/srep27035 (PMC4897644; doi:10.1038/srep27035)
Supplement: Supplementary Information [file srep27035-s1.pdf]

Supplementary Information

Metagenomic discovery of novel enzymes and biosurfactants in a slaughterhouse biofilm microbial community

Stephan Thies[1], Sonja Christina Rausch[1], Filip Kovacic[1], Alexandra Schmidt-Thaler[5], Susanne Wilhelm[1], Frank Rosenau[2], Rolf Daniel[3], Wolfgang Streit[4], Jörg Pietruszka[5,6], Karl-Erich Jaeger[1,6]*

[1] Institut für Molekulare Enzymtechnologie, Heinrich-Heine-Universität Düsseldorf, Jülich, Germany

[2] Zentrum für Peptidpharmazeutika, Universität Ulm, Ulm, Germany

[3] Institut für Mikrobiologie und Genetik, Abteilung für Genomik und Angewandte Mikrobiologie & Göttingen Genomics Laboratory, Georg-August-Universität Göttingen, Germany-

[4] Biocenter Klein Flottbek, Department of Microbiology and Biotechnology, University of Hamburg, Hamburg, Germany

[5] Institut für Bioorganische Chemie, Heinrich-Heine-Universität Düsseldorf, Jülich, Germany

[6] Institut für Bio- und Geowissenschaften IBG-1: Biotechnologie, Forschungszentrum Jülich GmbH, Jülich, Germany

This file includes:

Supplementary NMR data, Supplementary Figures S1 – S4 and Supplementary Tables S1 – S2

Supplementary NMR data

Chemical shifts for N-myristoyltyrosin protons and carbon atoms obtained from NMR experiments

NMR data of chemically synthesized N-myristoyltyrosin:
^{1}H-NMR (600 MHz, MeOD): δ (ppm): 0.9 (t, $^{3}J_{14',J13'}$=7.2 Hz, 3H, H-14‘), 1.29 (br, 22H, H-13’-H-3’), 1.5 (2t, $^{3}J_{3',\ 4'=}$ 7.2 Hz, $^{3}J_{2',3'}$=7.36 Hz, 2H, H-3’), 2.15 (t, $^{3}J_{2',3'}$= 7.36, 2H, H-2’), 2.84 (dd, $^{2}J_{5a,5b}$=13,75 Hz, dd, $^{2}J_{5a,5b}$=13.75 Hz, $^{3}J_{5a,6}$=4.64 Hz, 1H, H-5a), 3.11 (dd, $^{2}J_{5b,5a}$=13,75, $^{3}J_{5b,6}$ =4.64 Hz, 1H, H-5b, 4.61 (m, 1H, H-6), 6.69 (d, $^{3}J_{2,3}$=8.0 Hz, $^{3}J_{2'',3''}$=8.0 Hz, 2H, H-2, H-2’’), 7.04 (d, $^{3}J_{3,2}$=8.0 Hz, $^{3}J_{3'',2''}$=8.0 Hz, 2H, H-3, H-3’’).
^{13}C-NMR (150 MHz, MeOD): δ (ppm): 14.32 (1C, C-14’), 23.64 (1C, alkyl chain), 26.83 (1C, C-3’, 30.07, 30.41, 30.51 (3C, alkyl chain), 30.7 (2C), 30.74, 33.01 (2C alkyl chain), 36.75 (1C, C-2’), 37.57 (1C, C-5), 55.08 (1C, C-6), 116.97 (2C, C-2, C-2’’), 131.12 (2C, C-3, C-3’’), 157.26 (1C, C-1), 174.95 (1C, C-7), 176.05 (1C, C-1’).
NMR data of isolated biosurfactant:
^{1}H-NMR (600 MHz, MeOD): δ (ppm): 0.92(t, $^{3}J_{14',13'}$=7.2 Hz, 3H, H-14‘),1.31 (br, 22H, H13‘-H-3‘), 1.52 (2t, $^{3}J_{3',4'}$=7.4 Hz, $^{3}J_{2',3'}$= 7.4 Hz, 2H, H-3’), 2.18 (t, $^{3}J_{2',3'}$=7.4 Hz, 2H, H-2’), 2.86 (dd, $^{2}J_{5a,5b}$=14.1 Hz, dd, $^{2}J_{5a,5b}$=14.1 Hz, $^{3}J_{5a,6}$=4.4 Hz, 1H, H-5a), 3.13 (dd, $^{2}J_{5b,5a}$=14.1 Hz, $^{3}J_{5b,6}$=4.4 Hz, 1H, H-5b), 4.63 (m, 1H, H-6), 6.72 (d, $^{3}J_{2,3}$=8.7 Hz, $^{3}J_{2'',3''}$=8.7 Hz, 2H, H-2, H-2’’), 7.06 (d, $^{3}J_{3,2}$=8.7 Hz, 2H, H-3, H-3’’).
^{13}C-NMR (150 MHz, MeOD): δ (ppm): 14.67 (1C, C-14’), 23.76 (1C, alkyl chain, 26.95 (1C, C-3’), 30.16, 30.48, 30.61 (3C, alkyl chain), 30,78 (2C), 30.81, 33.10 (2C, alkyl chain), 36.86 (1C, C-2’), 37.69 (1C, C-5), 55.19 (1C, C-6), 116.19 (2C, C-2, C-2’’), 131.27 (2C, C-3, C-3’’), 157.3 (1C, C-1), 175.12 (1C, C-7), 176.21 (1C, C-1’).

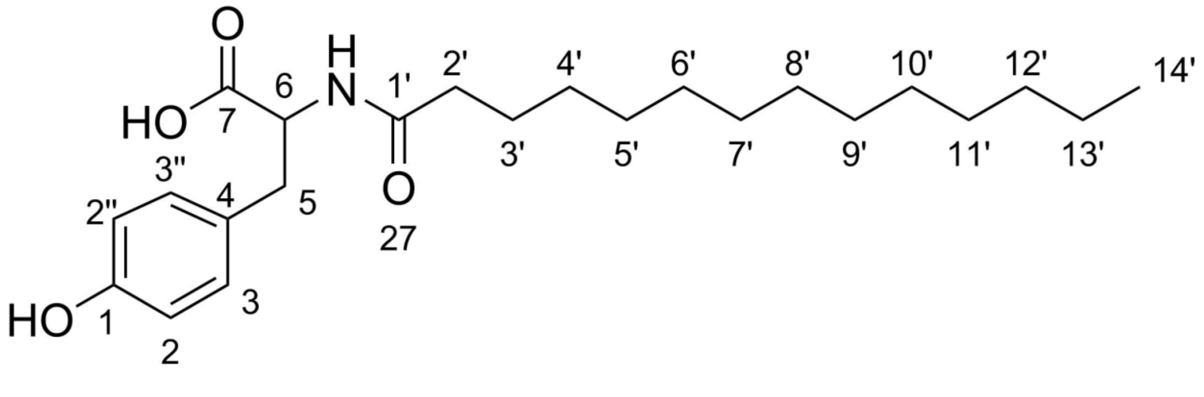

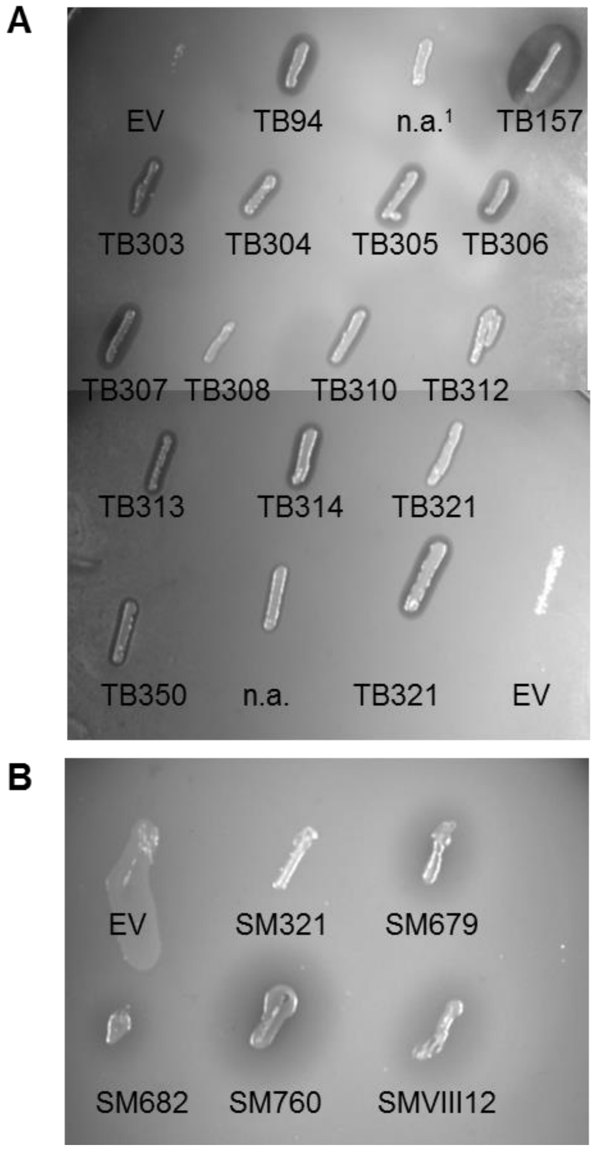

Figure S1: Phenotypic screening of clones from a slaughterhouse metagenomic library. *E. coli* DH10b clones expressing genes isolated from a slaughterhouse drain biofilm were grown on A) tributyrin LB-agar plates and B) skim milk LB-agar plates for detection of lipolytic and proteolytic activities, respectively. Clones active in the first screening on tributyrin and skim milk plates were restreaked and the results are shown here. Clones were designated as indicated in table S1. n.a. = non active clones.

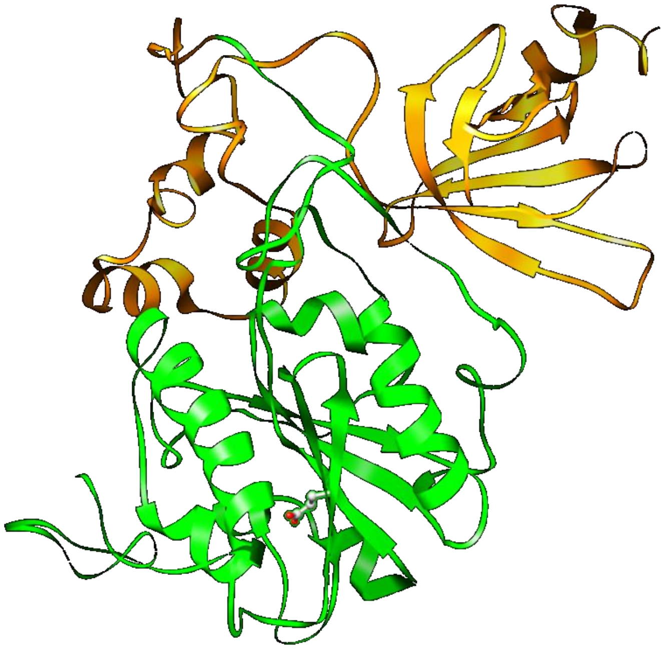

Figure S2: Homology model of Nas343. Unique two domain architecture of Nas343 (orf1 from eDNA fragment 343) as modelled with Phyre2 server. The model of Nas343 resembles the one of Nas354. In green is shown the N-terminal NAS domain modelled with the structure of FeeM from uncultured bacteria (PDB code 2G0B) and in orange is shown the C-terminal domain modelled with c-di-GMP-binding protein from *Pseudomonas aeruginosa* (PDB code 1YWU). The putative catalytic active site Glu103 residue is shown in ball-and-sticks presentation.

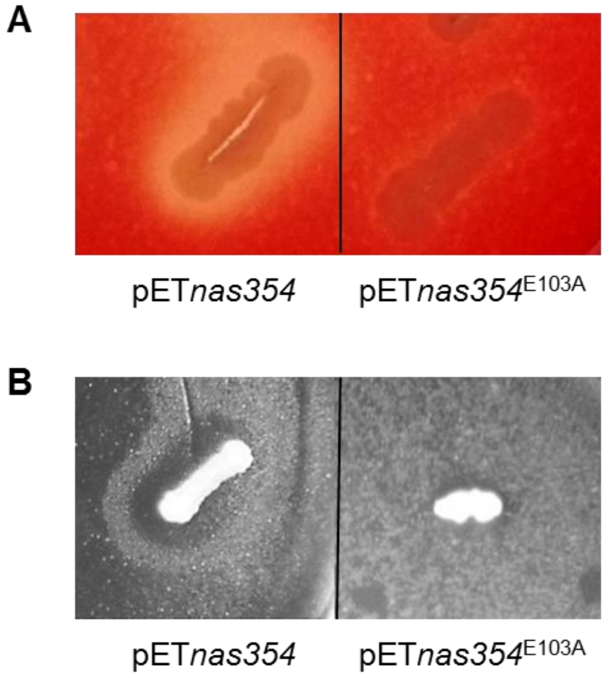

Figure S3: Mutation of the active site glutamate of Nas354 abolishes biosurfactant production. *E. coli* DH10b expressing Nas354 with the putative catalytic Glu103 replaced by alanine lack haemolytic activity on the blood-agar plates and surface activity in atomized oil assay indicating an essential role of Glu103 for the synthesis of N-acyltyrosine. The expression of *nas354* gene was induced by addition of 0.4 mM IPTG in the LB agar plates.

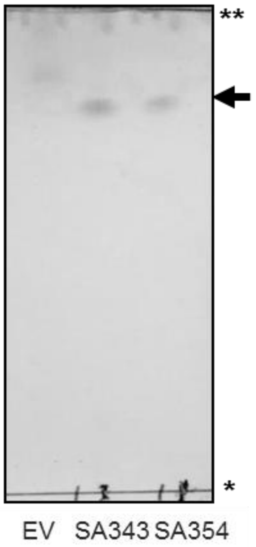

Figure S4: Identification of biosurfactant. A) TLC analysis of extracts obtained from culture supernatants of *E. coli* DH10b carrying pEBP*SA343*, pEBP*SA354* or pEBP18 (empty vector, EV). Cell free supernatants were precipitated with HCl followed by organic solvent extraction. TLC plates were stained with iodine vapour. The position of the isolated biosurfactant later identified as N-acyltyrosine is indicated. Start and solvent fronts are indicated by one and two asterisks respectively.

Supplementary tables

Table S1 Activities identified by functional screening

Activity	Screened clones	Positive clones	Unique clones
lipolytic	1.7×10^5	15	15
proteolytic	1.7×10^5	5	3
haemolytic+surfactant	1.7×10^5	2	2

Table S2: Bioactive clones identified in a slaughterhouse metagenomic library.

Clone[1]	Insert-size/Kb	ORF No.	start[2]	end[2]	Highest scoring BLAST hits[3]	score, coverage, identity[4]	Genbank accession[5]
SA343	3.3	1	244	1428	**LPLAT family, best hit hypothetical protein Daro_0269 [*Dechloromonas aromatica* RCB]**	300/58%/70%	CP000089
		2	2433	1588	peptidase S1 and S6 chymotrypsin/Hap [*Alicycliphilus denitrificans* BC]	273/57%/73%	CP002657
SA354	8.8				overall: *Sinorhizobium meliloti* Rm41 plasmid pSYMA complete sequence	6004/63%/86%	HE995407
		1	1622	36	putative monosaccharide ABC-transporter [*Sinorhizobium meliloti Rm41*]	1836/100%/83%	HE995407
		2	2905	1775	ABC-type transport system substrate binding [*Sinorhizobium meliloti SM11*]	1450/99%/88%	HE995407
		3	4868	3243	glucose-methanol-cholin Oxidoreductase [*Sinorhizobium meliloti* SM11]	2252/100%/88%	HE995407
		4	5537	4872	uracil-DNA glycosylase superfamily [*Sinorhizobium meliloti BL225C*]	629/90%/83%	HE995407
		5	6232	7416	**LPLAT, best hit hypothetical protein Daro_0269 [*Dechloromonas aromatica* RCB**]	271/59%/68%	CP000089
		6	8462	7557	peptidase S1 and S6 chymotrypsin/Hap [*Alicycliphilus denitrificans* BC]	300/71%/74%	CP002657
SM321	5.4	1	48	1781	**extracellular protease precursor [*Stenotrophomonas maltophilia* D457]**	711/88%/70%	HE798556
		2	1870	4233	**lysyl-endopeptidase 3 [*Lysobacter gummosus strain* UASM 402]**	751/63%/71%	KF738075
		3	5295C	4471C	asparaginase/glutaminase (aspG) [*Stenotrophomonas maltophilia* JV3/][*Xanthomonas campestris pv. campestris* ATCC 33913]	511/91%84%<br>533/54% 84%	CP002986<br>AE008922
SM679	5				overall: none complete; *Stenotrophomonas maltophilia JV3*, complete genome	502/49%/69%	CP002986
		1	4511C	3294C	general secretion pathway protein F [*Xanthomonas campestris pv. campestris* ATCC 33913]	100/58%65%	AE008922
		2	1119C	529C	general secretion pathway protein G [*Xanthomonas albilineans* GPE PC73]	125/60%/68%	FP565176
		3	1347	3101	**peptidase S8/S53 subtilisin kexin sedolisin [*Stenotrophomonas maltophilia JV3*]**	502/76%/79%	CP002986
SM760	3.5				overall: *Stenotrophomonas maltophilia JV3*, complete genome	66%	CP002986
		1	80	161	**peptidase S8/S53 subtilisin kexin sedolisin [*Stenotrophomonas maltophilia* K279a**]	515/76%/69%	CP002986
		2			general secretion pathway protein F	100/63%/65%	CP002986
TB94	5.5				overall: *Acidovorax* sp. KKS102 whole genome	5236/98%/84%	CP003872
		1	1	1272	inner-membrane translocator	1571/100%/87%	CP003872
		2	1365	3509	**putative D--3-hydroxybutyrate oligomer hydrolase lipoprotein transmembrane**	2412/98%/85%	CP003872
		3	3593	4768	ABC-type branched-chain amino acid transport systems, periplasmic component, extracellular ligand-binding receptor	1346/92%/87%	CP003872
		4	4837	5493	**predicted hydrolases or acyltransferases (alpha/beta hydrolase superfamily**	724/99%/84%	CP003872
TB157	6	1	310	1269	**lipolytic enzyme alpha/beta hydrolase fold [uncultured bacterium clone HZ11 (tribut. degrading activity) or *Sphingomonas wittichii* RW1**]	129/63%68%<br>107/23%72%	AY687451 or<br>CP000699
		2	2325C	1438C	**alpha/beta hydrolase fold protein [*Starkeya novella* DSM 506]**	563/91%/76%	CP002026

		3	3110C	2382C	2,4-dienoyl-CoA reductase [NADPH] /NADH:flavin oxidoreductase/NADH oxidase [*Burkholderia cenocepacia* H111 or *Paracoccus denitrificans* PD1222]	215/54% 73% 192/65%/68%	HG938371 or CP000490
TB303	11	1	2258C	216C	pyruvate flavodoxin/ ferredoxin oxidoreductase-like protein [*Sphingopyxis alaskensis* RB2256]	1362/88%/67%	CP000356
		2	2305	3261	**putative esterase Alpha/beta hydrolase fold-3 [*Sphingopyxis alaskensis* RB2256]**	315/87%/69%	CP000356
		3	3258	3734	Nudix hydrolase(phosphohydrolases) [*Sphingopyxis alaskensis* RB2256]	228/76%/74%	CP000356
		4	3742	4239	**dialkyl PEs hydrolase (dphB) gene [Uncultured bacterium clone 2]**	69.8/35%/69%	KC438416
		5	5178	5504	hypothetical protein [*Riemerella anatipestifer* RA-CH-2]	59/88%/65%	CP004020
		6	8269	8718	GAF-domain containing protein [*Flavobacteriaceae bacterium* 3519-10]	187/74%/72%	CP001673 6
		7	8715	9341	ribosomal RNA small subunit methyltransferase G [*Weeksella virosa* DSM 16922]	201/93%/69%	CP002455
		8	9438	10949	DNA gyrase subunit B (truncated) [*Weeksella virosa* DSM 16922]	1023/99%/73%	CP002455
		9	6928C	6337C	membrane protein *[Weeksella viros*a DSM 16922]	129/54%/70%	CP002455
TB304	3.5				overall: *Sphingopyxis alaskensis RB2256*, complete genome	1375/89%/77%	CP000356
		1	2072C	30C	pyruvate flavodoxin/ferredoxin oxidoreductase- like protein	1359/88%/77%	CP000356
		2	2119	3075	**lipolytic enzyme**	327/87%/69%	CP000356
		3	3072	3545	(truncated) RNA pyrophosphohydrolase	228/76%/74%	CP000356
TB305	4.5	1	2661	4151	**carboxylesterase, type B [*Sphingomonas wittichii* RW1]**	565/85%/70%	CP002008
		2	2275	1C	TonB-dependent receptor [*Caulobacter segnis* ATCC 21756]	334/91%/65%	CP002008
TB306	12.8	1	1C	402C	probable integrase *[Flavobacteriaceae bacterium* 3519-10]	581/100%/92%	CP001673
		2	373	870	hypothetical protein *[Flavobacteriaceae bacterium* 3519-10]	262/46%/64%	CP001673
		3	1148	1438	putative transcriptional regulator, probable addiction module antidote protein [*Cellulophaga algicola* DSM 14237]	235/98%/68%	CP002453
		4	1849	2130	transposase IS3/IS911 family protein [*Leadbetterella byssophila*] DSM 17132	136/61%/78%	CP002305
		5	2157	3038	transposase/integrase [*Leadbetterella byssophila* DSM 17132]	158/69%/66%	
		6	6261C	5833C	hypothetical proteine Phage-related lysozyme (muraminidase) [*Flavobacterium columnare* ATCC 49512]	87,8/55%/69%	CP003222 R
		7	8527	10017	**carboxylesterase, type B [*Sphingomonas wittichii* RW1]**	565/84%/70%	CP000699
		8	10041	10802	TonB-dependent receptor [*Caulobacter segnis* ATCC 21756]	226/84%/65%	CP002008
		9	10805	11683	oxidoreductase [*Bradyrhizobium* sp. ORS278]	361/99%/71%	CU234118
		10	119919	12308	ROK family protein	114/80%/69%	CP002102
TB307	4.8				overall: [*Acidovorax sp. KKS102*]	5225/99%/84%	CP003872
		1	193	1533	innermembrane translocator	1593/100%/86%	CP003872
		2	1626	3770	**putative D--3-hydroxybutyrate oligomer hydrolase lipoprotein transmembrane**	2385/98%/85%	CP003872
		3	3854	4807	ABC-type branched-chain amino acid transport systems, periplasmic component, extracellular ligand-binding receptor	1045/90%/87%	CP003872

TB308	4.5	1	1485	2087	flavoprotein WrbA [*Acidovorax ebreus* TPSY]	592/100%/82%	CP001392
		2	596	1366	**esterase, hypothetical protein (experimental evidance, homology: OsmC-like protein) [*Ramlibacter tataouinensis* TTB310]**	313/91%/70%	CP000245
		3	2823C	2416C	CsbD family protein [*Variovorax paradoxus EPS]*	86/44%/70%	CP002417
		4	3490C	2921C	phosphoribosylaminoimidazole-carboxamide formyltransferase-> IMPCH superfamily [*Delftia acidovorans* SPH-1]	993/99%/83%	CP000884
		5	4434C	3490C	bifunctional purine biosynthesis protein purH [*Alicycliphilus denitrificans* K601]	592/100%/83%	CP002657
TB310	3.8	1	1509	2531	**lipolytic enzyme, Alpha/beta hydrolase fold-3 protein/ Uncultured bacterium clone 2 dialkyl PEs hydrolase (dphB) gene [*Sphingopyxis alaskensis* RB2256]**	224/88%/67%<br>237/78%/68%	CP000356<br>KC438416
		2	1431C	148C	cyclopropane-fatty-acyl-phospholipid synthase [*Sphingopyxis alaskensis* RB2256]	230/79%/68%	CP000356
		3	2528	3004	putative (di)nucleoside polyphosphate hydrolase [*Sphingobium japonicum* UT26S]	212/84%/72%	AP010803
TB312	6.9	1	93	1676	metaphosphatase [*Ramlibacter tataouinensis* TTB310]	833/94%/73%	CP000245
		2	2558	3733	protein tyrosine/serine phosphatase [*Acidovorax citrulli* AAC00-1]	422/87%/78%	CP000512
		3	2559C	1747C	putative phytochrome sensor protein [*Alicycliphilus denitrificans* K601]	300/73%/68%	CP002657
		4	5294C	4301C	**triacylglycerol lipase [*Psychrobacter* sp. 7195]**	1563/100%/96%	AM229327
		5	6592	6924	(truncated) MatE efflux family protein, multidrug transporter [*Psychrobacter sp*. G]	351/99%/83%	CP006265
TB313	4.9	1	1	330	truncated thiolase [*Comamonas testosteroni* CNB-2]	298/98%/78%	CP001220
		2	364	2049	**carboxylesterase type B [*Variovorax paradoxus* EPS]**	315/66%/72%	CP002417
		3	3823	4912	truncated PAS domain containing protein/ methyl-accepting chemotaxis protein [*Acidovorax sp.* KKS102]	499/78%/74%	CP003872
TB314	3.2	1	1	486	**[*Moraxella* sp.] lipase**	504/98%/84%	X53869
		2	1539C	586C	**triacylglycerol lipase [*Psychrobacter* sp. 7195]**	1595/100%/97%	AM229327
		3	3086C	2763C	**[*Moraxella* sp.] lipase**	268/75%/84%	X53869
TB321	5.6				overall: [*Sphingopyxis alaskensis* RB2256], complete genom	2749/89%/85%	CP000356
		1	205	1137	**acyl glycerol lipase**	529/96%/74%	CP000356
		2	3341	4714	argininosuccinate lyase -> arginine biosynthesis	1602/98%/86%	CP000356
		3	4928	5609	diaminopimelate decarboxylase -> lysin biosynthesis	791/97%/88%	CP000356
		4	1174	2175	glycerol-3-phosphate dehydrogenase [NAD(P)+] 2	1115/89/85	CP000356
		5	2337	2639	hypothetical protein [*Sphingobium sp.* SYK-6]	59/42%/70%	AP012222
TB350	10.4	1	1035	1811	threonine dehydratase [*Stenotrophomonas maltophilia* D457]	383/78%/78%	HE798556
		2	1983	2612	conserved hypothetical protein [*Xanthomonas campestris pv. campestris str.* ATCC 33913,]	348/86%/79%	AE008922
		3	3407C	3414C	biotin biosynthesis protein BioC [*Pseudoxanthomonas suwonensis* 11-1]	237/30%/79%	CP002446
		4	5033C	4307C	**alpha/beta hydrolase fold, bioH [*Pseudoxanthomonas suwonensis* 11-1]**	288/95%/71%	CP002446

		5	6642C	5098C	**alkaline lipase [*Acinetobacter radioresistens*]**	235/30%/72%	AF073953
		6	7017	7715	exonuclease [*Acinetobacter oleivorans* DR1]	378/95%/73%	CP002080
		7	9575	9805	hypothetical protein [*Acinetobacter baumannii* ZW85-1]	129/86%/75%	AB811415
TB I H8	4.4	1	291	1679	**secretory esterase/lipase [*Acidovorax* sp. KKS102]**	1411/98%/83%	CP003872
		2	3005	4394	type III restriction protein res subunit -> PLCd superfamily [*Leptothrix cholodnii* SP-6]	1048/91%/82%	CP001013
		3	2905C	1793C	tRNA-specific 2-thiouridylase mnmA [*Alicycliphilus denitrificans* K601]	1254/96%/86%	CP002657

[1] First two letters of each clone indicate screening method by which clone was detected, SA = surface activity, SM = skim milk, TB = tributyrin.

[2] C = complement strand

[3] The best matching database sequence with the highest alignment score in BLAST output. Genes encoding putative lipolytic, proteolytic and hemolytic activities are printed in bold.

[4] The highest alignment score (Max score) from the given database sequence, the percentage of query covered by alignment to the database sequence, the highest percent identity (Max ident) of all query-subject alignments, as provided by NCBI BLAST (blast.ncbi.nlm.nih.gov).

[5] GenBank accession code of the best matched database sequence

Table S3: NCBI GenBank accession numbers of metagenomics clones.

Clone name	Genbank accession number
SA343_p	KM669715
SA354_p	KM669716
SM321_p	KM669717
SM679_p	KM669718
SM760_p	KM669719
TB157_p	KM669720
TB303_p	KM669721
TB304_p	KM669722
TB305_p	KM669723
TB306_p	KM669724
TB307_p	KM669725
TB308_p	KM669726
TB310_p	KM669727
TB312_p	KM669728
TB313_p	KM669729
TB314_p	KM669730
TB321_p	KM669731
TB350_p	KM669732
TB94_p	KM669733
TB_I_H8_p	KM669734
